# Effectiveness of continuous glucose monitoring in pregnant women with diabetes: randomised clinical trial

**DOI:** 10.1136/bmj.a1680

**Published:** 2008-09-25

**Authors:** Helen R Murphy, Gerry Rayman, Karen Lewis, Susan Kelly, Balroop Johal, Katherine Duffield, Duncan Fowler, Peter J Campbell, Rosemary C Temple

**Affiliations:** 1 Department of Diabetes and Endocrinology, Ipswich Hospital NHS Trust, Ipswich IP4 5PD; 2 Departments of Diabetes and Obstetrics, Ipswich Hospital NHS Trust; 3 Department of Obstetrics, Ipswich Hospital NHS Trust; 4 Elsie Bertram Diabetes Centre, Norfolk and Norwich University Hospital NHS Trust, Norwich; 5 Wellcome Trust Sanger Institute, Cambridge

## Abstract

**Objective** To evaluate the effectiveness of continuous glucose monitoring during pregnancy on maternal glycaemic control, infant birth weight, and risk of macrosomia in women with type 1 and type 2 diabetes.

**Design** Prospective, open label randomised controlled trial.

**Setting** Two secondary care multidisciplinary obstetric clinics for diabetes in the United Kingdom.

**Participants** 71 women with type 1 diabetes (n=46) or type 2 diabetes (n=25) allocated to antenatal care plus continuous glucose monitoring (n=38) or to standard antenatal care (n=33).

**Intervention** Continuous glucose monitoring was used as an educational tool to inform shared decision making and future therapeutic changes at intervals of 4-6 weeks during pregnancy. All other aspects of antenatal care were equal between the groups.

**Main outcome measures** The primary outcome was maternal glycaemic control during the second and third trimesters from measurements of HbA_1c_ levels every four weeks. Secondary outcomes were birth weight and risk of macrosomia using birthweight standard deviation scores and customised birthweight centiles. Statistical analyses were done on an intention to treat basis.

**Results** Women randomised to continuous glucose monitoring had lower mean HbA_1c_ levels from 32 to 36 weeks’ gestation compared with women randomised to standard antenatal care: 5.8% (SD 0.6) *v* 6.4% (SD 0.7). Compared with infants of mothers in the control arm those of mothers in the intervention arm had decreased mean birthweight standard deviation scores (0.9 *v* 1.6; effect size 0.7 SD, 95% confidence interval 0.0 to 1.3), decreased median customised birthweight centiles (69% *v* 93%), and a reduced risk of macrosomia (odds ratio 0.36, 95% confidence interval 0.13 to 0.98).

**Conclusion** Continuous glucose monitoring during pregnancy is associated with improved glycaemic control in the third trimester, lower birth weight, and reduced risk of macrosomia.

**Trial registration** Current Controlled Trials ISRCTN84461581.

## Introduction

Macrosomia, defined as infant birth weight on or above the 90th centile for sex and gestational age, remains the commonest complication of pregnancy in women with diabetes. Nationwide studies from the Netherlands, United Kingdom, and Denmark confirm that the risk of delivering a large for gestational age, or macrosomic, infant in women with type 1 diabetes ranges from 48.8% to 62.5%.^1^ ^2^ ^3^ Recent data confirm that women with type 2 diabetes have an equally high risk of delivering a macrosomic infant.^4^ ^5^ For mothers with diabetes, macrosomia leads to increased risk of perineal lacerations, complications in labour, and delivery by caesarean section.^6^ For the infants the risks of immediate complications are increased, including intracranial haemorrhage, shoulder dystocia, neonatal hypoglycaemia, jaundice, and respiratory distress^7^ ^8^ as well as the increasingly apparent longer term health risks of insulin resistance, obesity, and type 2 diabetes.^9^ ^10^

Major advances in the management of diabetes during pregnancy over the past 50 years have contributed to dramatic reductions in stillbirths and perinatal mortality but have not had a major impact on birth weight, with the risk of macrosomia persistently increased.^11^ Prepregnancy care is key to improved glycaemic control during the first two trimesters but does not reduce the risk of macrosomia, which we believe is more strongly associated with hyperglycaemia in later gestation.^12^ Data from the Netherlands suggest that near optimal glycaemic control during early pregnancy (84% of women had a mean HbA_1c_ level of 7.0% or less) failed to reduce the risk of macrosomia, present in 48.8% infants.^1^ This prompted us to consider new strategies, focusing on reducing postprandial hyperglycaemic spikes during the second and third trimesters. Observational data suggest a strong correlation between maternal postprandial glucose levels in the third trimester with an increased risk of macrosomia.^13^ ^14^ ^15^ ^16^ ^17^ Educational approaches incorporating additional glucose testing after meals to improve glycaemic control in late gestation have shown potential to reduce birth weight.^18^ ^19^ The optimal frequency and timing of home glucose testing during pregnancy is, however, unknown, with few women managing to carry out the 10 daily tests required to document most glucose excursions.^20^

Novel methods of continuous glucose monitoring provide up to 288 measurements a day.^21^ ^22^ Detailed data on the magnitude and duration of glucose fluctuations, particularly overnight and after meals, give unique insights into daily glycaemic control, which are especially valuable during the physiological changes of pregnancy.^22^ Additionally, continuous glucose monitoring provides patients with visual feedback on the consequences on glycaemia of factors such as diet, exercise, and insulin regimens, which can be harnessed as a powerful educational tool.

Despite promising pilot evaluations of continuous glucose monitoring^23^ ^24^ it is an expensive and relatively inconvenient tool, prompting the National Institute for Health and Clinical Excellence to call for randomised studies assessing its efficacy on clinically meaningful end points.^25^ Evidence of effectiveness on long term glycaemic control or diabetes related morbidity has not been established, with previous studies limited to the use of blood glucose or HbA_1c_ levels as surrogate markers of morbidity from diabetes. We evaluated the effectiveness of antenatal continuous glucose monitoring on maternal glycaemic control and pregnancy related morbidity—namely, birth weight and risk of macrosomia in the offspring of mothers with type 1 and type 2 diabetes. The preliminary phases of the trial and detailed data on glycaemia are described elsewhere.^22^

## Methods

We carried out a prospective, open label randomised controlled trial comparing current antenatal care using intermittent self monitoring of glucose levels from capillary blood obtained using the finger prick technique (standard care) with antenatal care using intermittent monitoring of capillary blood glucose plus continuous glucose monitoring. The study statistician used computer generated randomised numbers in blocks of 20, concealed in sealed envelopes. We did not use minimisation algorithms. Research nurses trained in accordance with good clinical practice guidelines provided the women with their group allocation.

### Sample size

We calculated that a sample size of 70 would give 80% power to detect a 40% reduction in macrosomia at P=0.05, based on a macrosomia rate of 60%. For a 50% reduction in risk for macrosomia, a sample size of 70 would give 95% power. Previous interventional studies of postprandial glucose monitoring using capillary blood suggested that this was feasible, and in view of the relative expense of continuous glucose monitoring we hypothesised that a sizeable effect would be required for it to be integrated into routine antenatal clinical care.

### Setting and participants

From September 2003-6 we carried out a randomised controlled trial at two secondary care diabetic antenatal clinics in the United Kingdom. Both were staffed by two consultant diabetologists; a consultant obstetrician; a midwife, nurse, and dietitian specialising in diabetes care; and rotating specialist registrars in obstetrics and diabetes.

We enrolled pregnant women aged 16-45 with type 1 and type 2 diabetes if they provided written informed consent and were willing to wear a continuous glucose monitor. Exclusion criteria were limited to severe medical or psychological comorbidity, and no women were excluded. Overall, 71 of 93 (76%) women who were consecutively approached agreed to participate, of whom 46 (65%) had type 1 diabetes and 25 (35%) had type 2 diabetes. No significant differences were found between the women who participated or declined in age, ethnicity, type or duration of diabetes, HbA_1c_ level or gestational age at booking, attendance for prepregnancy care, and folic acid supplementation. The participants were therefore representative of women attending our diabetes antenatal clinics. Figure 1 shows the reasons for declining participation.

**Figure fig1:**
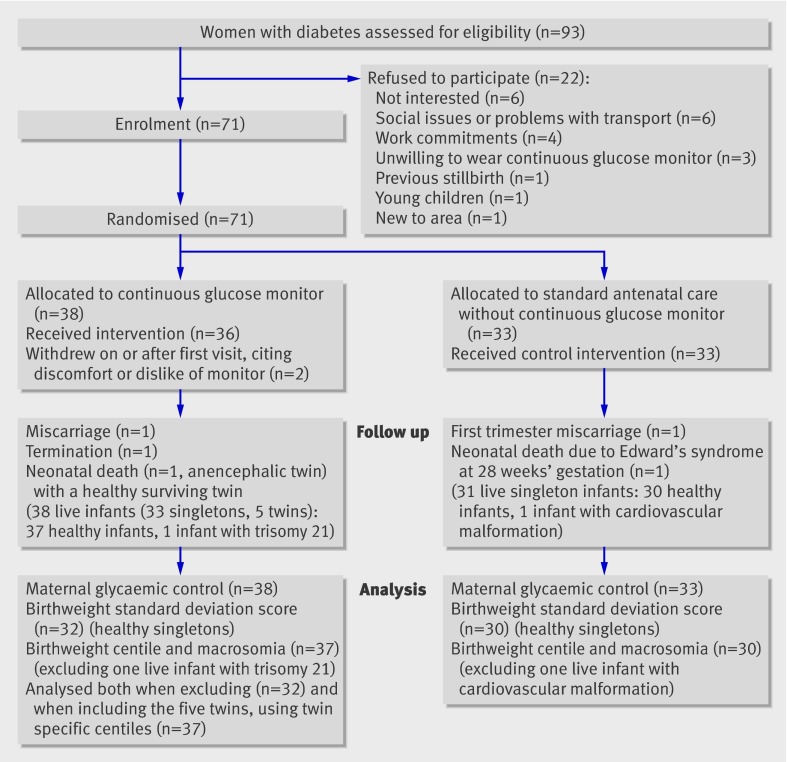
**Fig 1** Progression of women through trial

### Interventions

#### Study device

The continuous glucose monitor (CGMS Gold Medtronic-MiniMed, Northridge, USA) has a disposable subcutaneous device for sensing glucose and an electrode impregnated with glucose oxidase connected by a cable to a lightweight monitor. Interstitial glucose values in subcutaneous tissues, within a range of 2.2-22 mmol/l, are measured electrochemically every 10 seconds and an average value is stored every five minutes, providing up to 288 measurements a day. The system is recalibrated each time a capillary glucose measurement is entered, and women are advised to recalibrate the instrument at least four times a day. The accuracy, reliability, and measurement of glycaemic control by continuous glucose monitoring has been improved by recent modification of the sensor, allowing the device to be worn for up to seven days.^26^ ^27^

#### Study protocol

We offered supplementary continuous glucose monitoring for up to seven days at intervals of 4-6 weeks between 8 and 32 weeks’ gestation as our experience suggested that women had greater discomfort using continuous glucose monitoring in later pregnancy.^22^ Insertion of the sensor required an additional hospital visit (one week before the routine appointment at the diabetes clinic), and women were offered flexibility in scheduling both to assist compliance and to allow the sharing of limited equipment (three monitors per centre) among participants. Trained research nurses, with no clinical input at this visit, implanted the sensors into the upper outer buttock. Neither participants nor professionals had access to the glucose measurements during sensor use. The women removed the sensors after 5-7 days unless they experienced pain, discomfort, or technical problems, including sensor error. After one week the data were downloaded to a personal computer using software (Medtronic Com-station, version 1.7B) provided by the manufacturer.

At their scheduled clinic appointment the women handed their continuous glucose monitor or glucose meter in to the diabetes specialist nurse for downloading of the data. Downloaded data were printed out, with hard copies given to both the participants and the health professionals. When possible the women were given time to review the data with their support person in the waiting area before the clinical review. Women were asked firstly to note the proportion of time spent with glucose values within the target range, making efforts to focus attention on the positive aspects of each continuous or intermittent capillary glucose meter profile, before proceeding to identify patterns in hypoglycaemia and hyperglycaemia. They were advised to note down the likely causes for patterns that were out of range and to suggest possible solutions in terms of changes to diet, activity, and insulin dose. This was done in conjunction with the research team at the first visit and thereafter by the participants and their support person. The suggested therapeutic adjustments to diet, exercise, and insulin regimens were then discussed with the obstetric diabetes team, based on the combined intermittent capillary glucose and continuous glucose data for women allocated to continuous glucose monitoring or the intermittent capillary glucose data alone for women allocated to standard antenatal care.

The women were advised to measure blood glucose levels at least seven times a day and were provided with several targets: 3.5-5.5 mmol/l before meals, <7.8 mmol/l one hour after meals, and <6.7 mmol/l two hours after meals. The capillary blood glucose test results were verified using memory based reflectance meters. The women were seen every 2-4 weeks for up to 28 weeks, fortnightly until 32 weeks, and weekly thereafter, with assessments of fetal growth at 28, 32, and 36 weeks. Short acting insulin analogues were used before meals with intermediate acting insulin, long acting analogues, or pump therapy. The women with type 2 diabetes were treated with insulin before pregnancy or as soon as pregnancy was confirmed. Care provided at these centres both before and during pregnancy has been described, with no changes to routine clinical care during the study period.^12^ During labour and delivery we used intravenous infusions of glucose and insulin, with capillary blood glucose levels monitored hourly.

### Outcome measures

HbA_1c_ levels were measured once every four weeks and assayed using the aligned methodology (normal reference range 3.6-5.8%) of the diabetes control and complications trial in an accredited laboratory participating in the national external quality assurance programme. We calculated the birthweight standard deviation scores for only liveborn singletons using the British 1990 growth standards.^28^ We also calculated customised birthweight centiles using the open source gestation network (GROW) program,^29^ which uses maternal height, weight, ethnicity, and parity; neonatal sex; and gestational age, with twin standards used for twin pregnancies.^30^ Macrosomia is defined as a birth weight on or above the 90th centile, extremely large for gestational age as a birth weight on or above the 97.7th centile, and small for gestational age as a birth weight on or below the 10th centile. We obtained information on maternal characteristics, insulin doses, and HbA_1c_ levels from the hospital maternity records.

### Statistical analysis

We compared the baseline characteristics of the women using Fisher exact tests for discrete variables and *t* tests for continuous variables. For glycaemic control, we allocated the HbA_1c_ measurements to windows of four weeks, and compared the two arms using *t* tests. We also used a *t* test to compare the standard deviation scores for birth weight. As a result of the heavily skewed distribution of birth weight centiles, we compared these between the two arms using the non-parametric Wilcoxon rank sum test, and we used Fisher exact tests to compare the presence or absence of macrosomia. We used binomial tests for testing whether the proportion of small for gestational age infants was different from 10%. All analyses were done on an intention to treat basis, with statistical significance set at 5%. The data were analysed independently at the University of Cambridge using S-plus v7.0 (Insightful, Seattle, WA).

We excluded three infants in each group from the analyses of mean birth weight, birthweight centile, and birthweight standard deviation score as a result of miscarriage in the first trimester, neonatal death, and major malformation (fig 1). For the birthweight standard deviation scores we included only healthy singletons (n=62), with twins excluded as no comparative standard exists for twins. We did the analyses for birthweight centile both with twins (n=67), using the appropriate centile reference range for twins,^30^ and without twins (n=62).

## Results

Overall, 38 women were randomised to the continuous glucose monitoring intervention and 33 to standard antenatal care. The mean age of participants was 31.3 (SD 6.1) years, mean body mass index 28.1 (SD 7.4), and mean duration of diabetes 12.8 (SD 0.3) years (table 1). At booking the mean HbA_1c_ levels were 7.3% (SD 1.2%) and mean gestational age 9.2 (SD 2.7) weeks. Except for duration of diabetes, which was greater in women in the intervention group, no significant differences were found between the groups.

**Table 1 tbl1:** Baseline characteristics of pregnant women with pregestational diabetes allocated to antenatal care with continuous monitoring of blood glucose levels or to standard antenatal care only (control group). Values are numbers (percentages) unless stated otherwise

Characteristics	Continuous glucose monitoring (n=38)	Standard antenatal care (n=33)
Mean (SD) age (years)	30.2 (6.3)	32.5 (5.9)
Diabetes type:		
Type 1	28 (74)	18 (55)
Type 2	10 (26)	15 (45)
Mean (SD) duration of diabetes (years)*	15.2 (11.0)	10.0 (8.8)
Primiparous	16 (42)	11 (33)
Ethnicity:		
White European	34 (89)	29 (88)
Asian	3 (7.9)	3 (9.1)
Other	1 (2.6)	1 (3.0)
Mean (SD) body mass index (kg/m^2^)	27.9 (7.0)	28.4 (8.1)
Mean (SD) HbA_1c_ level at booking (%)	7.2 (0.9)	7.4 (1.5)
Mean (SD) gestational age at booking (weeks)	9.4 (2.3)	9.0 (3.0)
Prepregnancy care	24 (63)	18 (55)
Folic acid at booking	33 (87)	27 (82)
Microvascular complication	7 (18.4)	3 (9.7)
Smoker	5 (13.1)	4 (12.1)

*P=0.03.

### Continuous glucose monitoring

The continuous glucose monitor was generally well tolerated. No skin infections occurred although mild erythema and inflammation were often seen around the insertion site of the sensor. Two pregnancies in intervention women ended prematurely (one miscarriage and one termination). The mean number of periods for continuous glucose monitoring in the remaining 36 women was 4.2 (range 0-8), with 29 of the women (80%) wearing the monitor at least once per trimester. One woman who experienced pain after insertion of the sensor withdrew from the study and another declined to participate further after the first continuous glucose profile had been downloaded. Reasons cited for reduced use of the continuous glucose monitor were discomfort when wearing the sensor, transport, and difficulties with bathing.

### Glycaemic control

Although the HbA_1c_ level was consistently lower in the intervention arm no statistical difference was found in mean levels between the two groups at booking or throughout the first two trimesters (fig 2). Differences between the two arms began to emerge between 28 and 32 weeks’ gestation: mean HbA_1c_ levels in the intervention arm were 6.1% (SD 0.6) compared with 6.4% (SD 0.8) in the control arm, with a trend towards but not reaching statistical significance (P=0.1). In later pregnancy, at 32-36 weeks’ gestation, a further reduction in HbA_1c_ levels was seen in the intervention arm but no further reductions in the control arm—a difference in mean HbA_1c_ levels of 0.6% between groups: 5.8% (SD 0.6) in intervention arm compared with 6.4% (SD 0.7) in control arm (P=0.007).

**Figure fig2:**
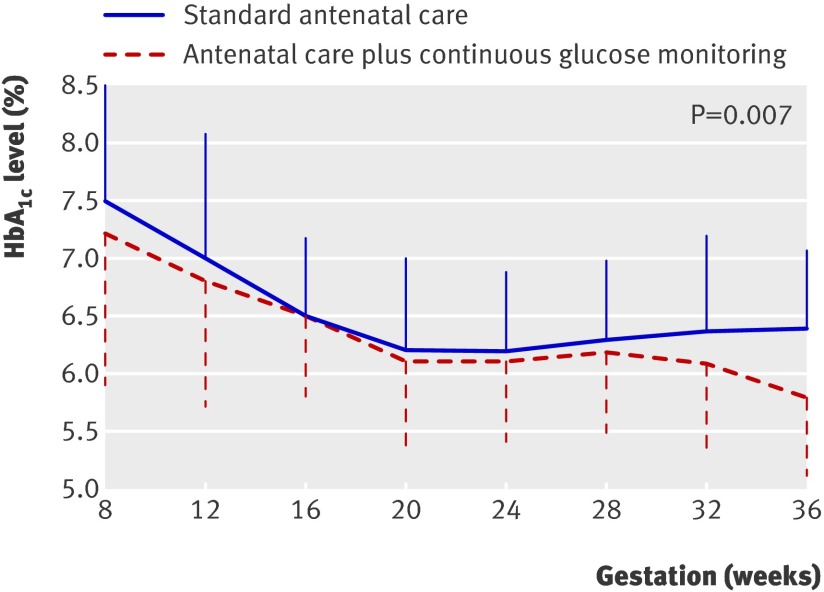
**Fig 2** Mean HbA_1c_ levels every four weeks in women receiving standard antenatal care (n=33) or antenatal care plus continuous glucose monitoring (n=38). Vertical lines are standard deviation at each time point

### Pregnancy outcomes

From 71 pregnancies there were 69 living infants (table 2). Two women miscarried in the first trimester (one miscarriage in each arm) and one woman had a termination at 19 weeks as a result of trisomy 21. Two early neonatal deaths occurred (one in each arm); a twin pregnancy with the death of one twin at 34 weeks’ gestation (anencephaly) and another at 28 weeks’ gestation (Edward’s syndrome). Two sets of twins plus the twin of the anencephalic infant resulted in five healthy twins (all offspring of women in the intervention arm). Two living infants had malformations, one cardiovascular (control arm) and one chromosomal (trisomy 21; intervention arm).

**Table 2 tbl2:** Pregnancy outcome in women with pregestational diabetes allocated to antenatal care plus continuous glucose monitoring or to standard antenatal care only (control group). Values are percentages (numbers) unless stated otherwise

Variable	Continuous glucose monitoring (n=38)	Standard antenatal care (n=33)	P value
No of first trimester miscarriages	1	1	1.0
No of terminations	1	0	1.0
Neonatal death*	1	1	1.0
No of live singletons	38	31	
No of live singletons with malformation	1 chromosomal†	1 cardiovascular	1.0
No of women with pre-eclampsia	2	0	0.5
No of twins‡	5	0	0.5
Delivery mode§:			
Vaginal	29 (11)	39 (12)	0.4
Elective caesarean	42 (16)	20 (6)	0.07
Emergency caesarean	29 (11)	43 (13)	0.3
Neonatal morbidity:			
Preterm delivery <37 weeks	16 (6)	19 (6)	0.8
Admission to neonatal care unit	24 (9)	19 (6)	0.8
Neonatal hypoglycaemia	8 (3)	17 (5)	0.5
Mean (SD) gestational age at delivery (weeks)	37.6 (1.3)	37.5 (1.5)	0.8
Mean (SD) birthweight standard deviation score¶	0.9 (1.0)	1.6 (1.4)	0.05
Mean (SD) birth weight (g)**	3340 (760)	3630 (500)	0.07
Median birthweight centile**	69	93	0.02
Macrosomia (≥90th centile)	35 (13)	60 (18)	0.05
Extremely large for gestational age (≥97.7th centile)	14 (5)	30 (9)	0.1
Small for gestational age (≤10th centile)	11 (4)	0	0.1

*Twin pregnancy with death of anencephalic twin and healthy surviving twin.

†Trisomy 21.

‡Two further sets of twins were delivered to mothers in intervention arm resulting in five healthy live twins.

§For comparison between elective and emergency caesarean section rates between groups, P=0.08.

¶Scores calculated only for 62 healthy living singletons (32 in intervention arm, 30 in control arm).

**Calculations were done for 67 of 69 healthy living infants (37 in intervention arm, 30 in control arm), after excluding one infant from each group as a result of congenital or chromosomal malformation. Twins are included, with centiles calculated using specific twin standards. Difference in birthweight centiles remained significant (P=0.04) when twins were excluded.

Two thirds of deliveries were by caesarean section with no difference between groups in the mean gestational age at delivery. No differences were found in rates of vaginal delivery compared with operative delivery but a trend was towards reduced emergency caesarean in the intervention group (P=0.08). Neonatal morbidity was relatively uncommon, with 17.4% preterm deliveries (n=12), 21.7% of infants admitted to neonatal care (n=15), and 11.6% of infants with hypoglycaemia (n=8). No significant differences were found between the groups.

### Birth weight

Compared with healthy singletons of women in the control group (n=30), those of women in the intervention group (n=32) had decreased mean birthweight standard deviation scores (0.9 *v* 1.6; effect size 0.7 SD, 95% confidence interval 0.0 to 1.3; P=0.05). Table 2 shows the mean birthweight centiles and median customised birthweight centiles for 67 of 69 healthy living infants. The median birthweight centiles were 69% in the intervention group compared with 93% in the control group (P=0.02), with twins included using a twin specific centile reference range.^30^ The difference in birthweight centiles remained significant after exclusion of the five twins (P=0.04).

In total, 31 of 67 (46%) infants were macrosomic, of whom 23 were born to mothers with type 1 diabetes (74%) and eight to mothers with type 2 diabetes (26%). Thirteen of 37 (35%) infants in the intervention arm were macrosomic compared with 18 of 30 (60%) in the control arm: odds ratio for reduced risk of macrosomia 0.36 (95% confidence interval 0.13 to 0.98); P=0.05. Also fewer extremely large for gestational age infants were born to mothers in the intervention arm than control arm: 5/37 (13.5%) *v* 9/30 (30%). This difference did not reach statistical significance (P=0.13). The two largest infants born to mothers in the intervention arm (5.39 kg and 4.07 kg, with birthweight centiles ≥97.7 and birthweight standard deviation scores >3.5, fig 3) were born to mothers who withdrew from the intervention (included in the intention to treat analysis).

**Figure fig3:**
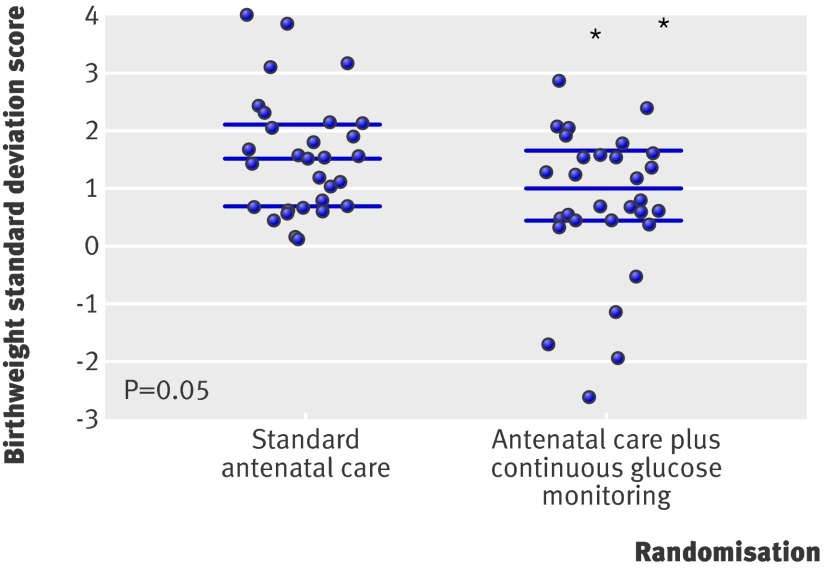
**Fig 3** Distribution of birthweight standard deviation scores for 62 healthy living singletons of mothers in continuous glucose monitoring arm (n=32) or standard antenatal care arm (n=30). *Infants of mothers who withdrew from intervention arm (included in intention to treat analysis). Thick lines indicate medians and thin lines interquartile ranges

One infant of a mother in the intervention arm had a birthweight standard deviation score below −2 (that is, below the 95% confidence interval) compared with infants of mothers in the control arm (fig 3). This girl was delivered at gestational age 34 weeks and six days to a primiparous mother (type 1 diabetes for four years) with severe pre-eclampsia and weighed 1530 g. On the basis of the birthweight centile definition of small for gestational age (birth weight ≤10th centile) four of 37 (10.8%) infants of women in the intervention arm (including the girl born to a pre-eclamptic mother) were small for gestational age compared with 0 of 30 infants born to women in the control arm (P=0.1). One infant was born to a mother with documented retinopathy and longstanding type 1 diabetes (21 years’ duration), and the remaining two to Asian women with type 2 diabetes with ethnically adjusted birthweight centiles exactly on the 10th centile, both of whom were clinically well and did not require neonatal care. By definition, 10% of infants would be expected to be on or below the 10th centile for birth weight. Formal testing showed that the number of small for gestational age infants in the intervention arm was no different from that expected (11% *v* 10%; P=1.0), whereas in the control arm, if anything fewer babies than expected were below the 10th centile (0%; P=0.07).

## Discussion

Our results suggest that the use of supplementary continuous glucose monitoring as an educational tool during pregnancy is associated with improved maternal glycaemic control. The results were clinically and statistically significant, as the infants of mothers in the intervention arm (continuous glucose monitoring) had a lower birth weight and a reduced risk of macrosomia than those of mothers in the control arm (standard antenatal care).

The observed differences in maternal HbA_1c_ levels, reflecting mean blood glucose levels over the preceding 4-6 weeks, began to emerge at 28 weeks but did not reach statistical significance until after 32 weeks’ gestation. Earlier studies of postprandial finger prick testing also found better glycaemic control in late gestation, with improvements in blood glucose levels during the second and third trimesters and in HbA_1c_ levels before delivery.^19^ ^31^ That HbA_1c_ levels do not improve until the third trimester may reflect the physiological delay between improved glycaemic control and clearance of glycated haemoglobin from the circulation. A learning curve also exists for women and health professionals when using continuous glucose monitoring to optimise lifestyle and therapeutic management, which in our experience takes several consultations. Indeed, we consistently observed that improvements in glycaemic profiles were achieved gradually over pregnancy, rather than after one consultation (data not shown). This emphasises the importance of using the data from continuous glucose monitoring as a tool to facilitate patient education and shared problem solving, and the necessity to reinforce the benefits over several visits. We have previously shown the influence of prepregnancy care on early glycaemic control and hypothesise that initiating continuous glucose monitoring earlier in pregnancy—ideally before conception—may help to achieve an earlier impact on glycaemic control.

Several possible explanations may contribute to the observed improvements in maternal and neonatal outcomes. Generally, in standard antenatal care women measure their blood glucose levels before and after meals, variably at intervals of one or two hours, with only the most motivated achieving the minimum of 10 daily tests required to document fluctuations in glucose levels.^20^ The continuous glucose monitoring profiles obtained in this study yielded maximal information about the frequency, magnitude, and duration of glucose excursions. We believe that the increased scrutiny of glucose excursions facilitated by continuous glucose monitoring may have contributed to increased motivation and compliance with self management, in particular glucose testing after meals among women in the intervention group. The visual impact of continuous glucose monitoring rapidly focused clinical input towards reducing hyperglycaemic spikes, which are less apparent from a patient diary or glucose meter download but essential if seeking to limit the transfer of glucose to a fetus. The continuous glucose monitoring data also provided feedback on the adequacy of prandial and basal insulin levels, day to day variability in diet, physical activity, and management of hypoglycaemia, with positive feedback to re-enforce optimal self management. Although women in the intervention arm had had diabetes for longer than women in the control arm this would not be expected to contribute to improvement in glycaemic control.

The relative contribution of hyperglycaemia compared with obesity in the development of macrosomia is controversial^32^ but as we adjusted the birthweight centiles for maternal height and weight, and baseline maternal body mass indices were similar in both groups, the observed differences in birth weight are most likely attributable to the improvements obtained in glycaemic control. Admittedly, HbA_1c_ levels are markers of average glycaemic control and do not provide detailed insight into hyperglycaemic spikes, implicated in the pathogenesis of macrosomia. The absence of comparative blinded continuous glucose data in women who received standard antenatal care means that we can only speculate on more complex differences in glycaemia between the groups.

Although we cannot fully exclude the possibility that tight glycaemic control contributed to fetal growth restriction, only one infant of a mother in the intervention arm had a birthweight standard deviation score below −2. Four infants of mothers in the intervention arm and no infants of mothers in the control arm were born small for gestational age. Ten per cent of infants would of course be expected to have a birthweight centile of 10% or less, and four of 37 (11%) is not significantly different from this prediction (P=0.1). Indeed, the most strikingly apparent difference in the number of infants who were small for gestational age between the two arms is the absolute absence of such infants of mothers in the control arm, reflecting the overall trend towards larger babies. Significant neonatal morbidity was, however, relatively uncommon in both groups, with only 17% preterm deliveries and 22% of infants admitted to neonatal care compared with 37% and 56% of infants nationally.^4^

### Strengths and limitations of the study

This trial combined recent technological advances in glucose monitoring with a pragmatic educational approach, suitable for widespread implementation in real life clinical settings. It fills a gap identified by the National Institute for Health and Clinical Excellence for more research into continuous glucose monitoring and the need for data not only on interim glucose and HbA_1c_ levels but also on diabetes related morbidity, including pregnancy and neonatal outcomes. Furthermore, the randomised design was robust, with high recruitment and retention rates. None of our exclusion criteria were applied, such that 76% of women participated, and despite the moderate inconvenience of wearing a glucose sensor, 80% of women used continuous glucose monitoring at least once per trimester. Only minimal equipment was required, with three continuous glucose monitors, costing about £1500 (€1889; $2693) each per centre—one monitor for every six pregnancies and four glucose sensors (about £40 each) per pregnancy. Carrying out the trial in a routine clinical setting with judicious use of resources and equipment is key to the generalisability and replicability of the intervention.

Some limitations should also be considered. Firstly, the women were predominantly of white European ethnicity, which may limit applicability to women from other cultures and ethnic backgrounds. Secondly, health professionals were not blinded and therefore we cannot fully exclude the possibility of bias in clinical management. Every effort was, however, made to standardise antenatal contacts between groups, with no difference in the frequency or duration of clinical appointments, dietary advice, obstetric input, or fetal surveillance, and no clinical input during attachment of the continuous glucose sensor. Thirdly, differences in maternal characteristics, with longer duration of diabetes in the intervention group, perhaps contributed to some of the effect on infant outcomes, although would not explain the improvements in glycaemic control. Fourthly, the number of women studied is small and larger multicentre trials are required to assess fully the many factors implicated in fetal growth and to document the true costs and benefits of the intervention.

### Putting the study in context

Several pilot studies have reported on the potential of continuous glucose monitoring to assist in adjustment of the insulin dose during pregnancy, but our randomised intervention study showed efficacy in maternal glycaemic control. Outside pregnancy, randomised studies have shown improvements in interim blood glucose excursions and HbA_1c_ levels associated with continuous glucose monitoring,^33^ but no studies have shown improvements in diabetes related complications or morbidity. Our study has filled a gap in the evidence base, as recognised by the National Institute for Health and Clinical Excellence,^25^ suggesting that new technologies can be used to reduce the risk of diabetes related complications in pregnancy.

The observed reductions in birth weight are particularly important in view of the widespread prevalence of macrosomia, affecting over half of all infants born to mothers with diabetes in the United Kingdom. Other advances, including insulin delivery by continuous subcutaneous infusion, have failed to reduce the risk of macrosomia. A recent Cochrane review found a significant increase in birth weight associated with pump therapy (weighted mean difference 220.56 g).^34^ In our experience insulin pumps remain limited by user input, particularly in the frequency of blood glucose testing after meals, following physical activity and correction of hypoglycaemia. As most of the women in our study were using multiple daily injections we suggest that further study of continuous glucose monitoring in women treated by pump is indicated, to determine the effectiveness in this group.

### Implications

These results show that continuous glucose monitoring during routine antenatal care can provide added benefits to the pregnancy outcomes for women with diabetes and their infants. Additional benefits on intention to treat analysis were a reduction in HbA_1c_ levels in the third trimester and reduced birth weight and risk of macrosomia. The consequences of birth weight are not limited to immediately apparent delivery injuries, which can be prevented by caesarean section, as infants born large for gestational age have an increased longer term risk of insulin resistance, obesity, and type 2 diabetes. The widespread prevalence and lasting effects of maternal hyperglycaemia suggest that this should be considered a potentially important target for public health strategies, aiming to reduce the burden of obesity in childhood.

Although rates of macrosomia were reduced in women using continuous glucose monitoring, they remain 3.5 times higher than the general maternity population. This suggests that despite continuous glucose monitoring helping women to improve average glycaemic control in late pregnancy, it is still inadequate for achieving optimal day to day glucose control and birth weights comparable with the background population. Perhaps newer generation devices for real time continuous glucose monitoring will offer the potential for a more rapid impact on glycaemic control earlier in pregnancy. Advances in computational approaches for calculating postprandial glucose fluxes^35^ and the development of closed loop systems^36^ may offer additional tools to aim for further reduction in risk of macrosomia in future decades.

We have shown that supplementary continuous glucose monitoring provided in a routine clinical setting leads to better glycaemic control and reduced risk of macrosomia. If confirmed by other studies these data have important implications for the antenatal management of women with diabetes as well as the immediate and longer term health of their infants.

**What is already known on this topic**

Continuous glucose monitoring is a potentially beneficial educational tool, with benefits on glycaemic control
NICE have called for research during pregnancy, to focus on pregnancy complications and infant outcomes

**What this study adds**

Continuous glucose monitoring is associated with improved maternal glycaemic control and reduced infant birth weight and risk of macrosomia
The beneficial effects on pregnancy complications potentially offer longer term health benefits for the infants of mothers with diabetes

## Notes

We thank the women who participated, Medtronic UK for donating study equipment, and coinvestigators Katharine Stanley, Sue Land, Nicoletta Dozio (Norwich UK), and Damian Morris (Ipswich UK) for comments on the manuscript.

Ethical approval: Suffolk and Norfolk local research ethics committees.

Provenance and peer review: Not commissioned; externally peer reviewed.

## Notes

**Cite this as: ***BMJ* 2008;337:a1680
